# Evolutionary conservation and over-representation of functionally enriched network patterns in the yeast regulatory network

**DOI:** 10.1186/1752-0509-1-1

**Published:** 2007-01-08

**Authors:** Ofer Meshi, Tomer Shlomi, Eytan Ruppin

**Affiliations:** 1 School of Computer Science, Tel-Aviv University, Tel-Aviv, Israel; 2 School of Medicine, Tel-Aviv University, Tel-Aviv, Israel

## Abstract

**Background:**

Localized network patterns are assumed to represent an optimal design principle in different biological networks. A widely used method for identifying functional components in biological networks is looking for network motifs – over-represented network patterns. A number of recent studies have undermined the claim that these over-represented patterns are indicative of optimal design principles and question whether localized network patterns are indeed of functional significance. This paper examines the functional significance of regulatory network patterns via their biological annotation and evolutionary conservation.

**Results:**

We enumerate all 3-node network patterns in the regulatory network of the yeast *S. cerevisiae *and examine the biological GO annotation and evolutionary conservation of their constituent genes. Specific 3-node patterns are found to be functionally enriched in different exogenous cellular conditions and thus may represent significant functional components. These functionally enriched patterns are composed mainly of recently evolved genes suggesting that there is no evolutionary pressure acting to preserve such functionally enriched patterns. No correlation is found between over-representation of network patterns and functional enrichment.

**Conclusion:**

The findings of functional enrichment support the view that network patterns constitute an important design principle in regulatory networks. However, the wildly used method of over-representation for detecting motifs is not suitable for identifying functionally enriched patterns.

## Background

Complex biological functions are performed by the integrated activity of functional modules consisting of highly interacting cellular components [1,2]. Network motifs are localized patterns of interconnections that occur at significantly higher numbers than in randomized networks and thus may represent components of functional modules [3]. Motifs identified in the transcriptional regulatory network of the bacteria *E. coli *were found to have important roles in information processing performed by the network and were used to obtain a compact representation of the network [4]. Specifically, a motif called "feed-forward loop" found in transcriptional regulatory networks was shown to have an important role in the regulation of genes in response to persistent stimuli in contrast to transient signals [4,5]. Superfamilies of various biological and other networks were identified to have similar local structure based on the significance profiles of network patterns [6]. Overall, network motifs have become a widely used method for identifying functionally significant network components.

A number of recent studies suggest that over-represented network patterns, i.e. network motifs, may not necessarily have functional significance: **(i) **Network motifs are found by testing a "random null hypothesis", comparing the abundance of patterns in an observed network with those found in an ensemble of randomized networks [3]. It was recently claimed that an ill-posed null hypothesis may lead to the false identification of significant network patterns, as such a randomization process may not correctly represent naturally evolved networks [7,8]. For example, it was claimed that the network motifs found in the neural-connectivity network of the nematode *C. elegans *may be the result of using a null hypothesis which does not account for localized aggregation of neural connections. **(ii) **Another recent work [9] has claimed that localized network patterns may not necessarily reflect evolutionary selection of functional components as the density of the patterns may be determined by the global structure of the network, which is characterized by its degree distribution and clustering coefficient. **(iii) **Further work [10] claims that network motifs do not seem to be of evolutionary or functional prominence. They compare the evolutionary conservation of motifs to that of randomly chosen network patterns and conclude that motifs are not subject to any particular evolutionary pressure acting for their preservation. A close examination of motif occurrences in well-studied pathways serving specific biological functions is then employed to show that motifs do not play a central regulatory role.

These results call for a reexamination of two fundamental questions concerning network patterns' analysis: **(i) **Do localized network patterns constitute an essential design principle of the underlying network? **(ii) **Is over-representation of network patterns a suitable measurement of evolutionary pressure and thus of functional significance?

Aiming to answer these questions, we conduct a large-scale analysis of functional enrichment and evolutionary conservation of all 3-node patterns in different dynamic regulatory networks in the yeast *S. cerevisiae*. Dynamic regulatory networks are subsets of the static regulatory network which are active under specific cellular conditions. A recent study [11] has uncovered large changes in the underlying network architecture between the static regulatory network of *S. cerevisiae *and "dynamic" subsets of this network. Structural properties of several dynamic networks were studied for 5 different cellular conditions: (i) cell cycle, (ii) sporulation, (iii) diauxic shift, (iv) DNA damage, and (v) stress response [11].

Functionally enriched occurrences of network patterns in these dynamic networks may represent important functional modules reflecting essential design principles. To measure functional enrichment we define a *mean functional enrichment *score which is based on the genes' GO annotation. Extending the work of [10] who studied the function of over-represented network patterns within the context of a small number of well known biological modules, our analysis examines the function of an ensemble of network patterns using large-scale annotation data. A previous study by [12] claimed that over-represented network patterns are functionally enriched without considering the enrichment of the non over-represented patterns as a background model. To determine that a specific pattern is functionally enriched we take a different approach and compare the enrichment of a pattern to that of the remaining patterns.

To measure evolutionary conservation of network patterns we generalize the standard scores of conservation of single genes to 3-node patterns via two conceptually distinct ways:

1. **"bag of nodes" **– inspecting the conservation scores within each occurrence induced by each 3-node pattern. We define the *mean conservation score *for each 3-node pattern as the mean conservation scores of all genes that form the pattern's occurrences (Methods).

2. **"coherency" **– inspecting the coherency of conservation scores within each occurrence induced by the 3-node pattern. Genes that together form a functional component are expected to be coherently conserved through evolution. We define the *conservation coherency score *as the percentage of the pattern's occurrences with coherent conservation, (i.e. occurrences with all genes in an occurrence having the same conservation level; Methods).

A similar definition for conservation coherency has been used in [13], where they study evolutionary conservation of network patterns in the protein-protein interaction network of the yeast *S. cerevisiae*. Another recent study by [10] has also measured the coherency of evolutionary conservation of interaction patterns in an integrated network of *S. cerevisiae *comprising transcriptional and protein-protein interaction data. They define a "fragility" score for a pattern's occurrence that reflects the tendency of its constituting genes to be uniformly present or absent in an ensemble of 4 related organisms. Our conservation coherency score is different as it is based on a large-scale prediction of the presence of genes in ancestral species of *S. cerevisiae *(Methods).

Applying these measures to various dynamic networks, we examine whether there are functionally enriched patterns that may represent specific regulatory mechanisms, and examine their evolutionary conservation. Finally, we turn to answer the second question, i.e. whether over-representation of network patterns is a good indicator of their functional significance.

## Results and Discussion

### Occurrences and Over-Representation of 3-Node Patterns

**Figure 1:**
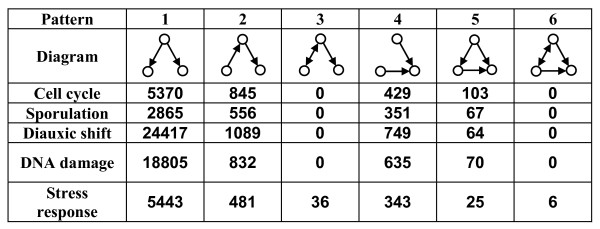
Number of occurrences of 3-node patterns in 5 dynamic regulatory networks. Pattern 1 has the highest number of occurrences in all dynamic networks, while pattern 6 has very few or no occurrences at all.

We searched for all occurrences of all 13 possible 3-node patterns in all dynamic networks and found that only 6 patterns occurred in at least one network (Methods, Figure 1). Pattern 6 (representing a feed-forward loop with an additional bidirectional interaction) was found only in the stress-response network with six occurrences, which is too few to detect significant functional enrichment or conservation measures and was thus excluded from further analysis. Pattern 3 was also found only in the stress-response network with 36 occurrences, suggesting that both patterns 3 and 6 may represent functions that are specific only to stress-response. Pattern 1, representing a transcription factor regulating two target genes, is significantly more common than the remaining patterns, in all dynamic networks.

**Figure 2:**
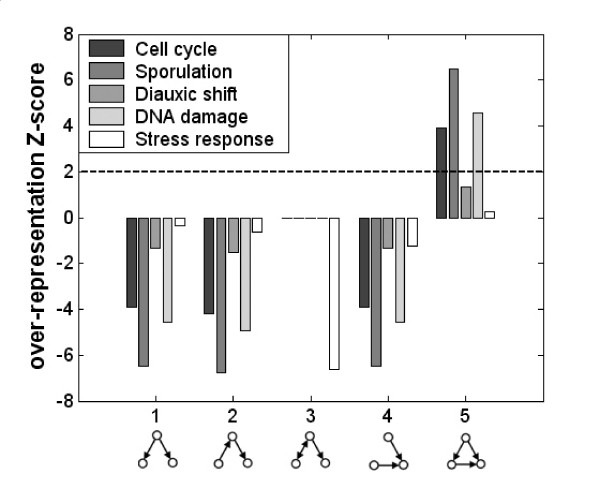
Over-representation of 3-node patterns in each of the different dynamic regulatory networks. Pattern 5 (feed-forward loop) is over represented in 3 out of 5 dynamic networks (Z-score > 2). No other pattern is over represented in these networks.

We computed the over-representation scores for all patterns in each dynamic network and found that pattern 5 (feed-forward loop) is over-represented (Z-score *> *2) in the cell-cycle, sporulation and DNA-damage networks (Methods, Figure 2). The over-representation of the feed-forward loop is in agreement with previous results in the static regulatory networks of the bacteria *E. coli *and the yeast *S. cerevisiae *[4,14].

### Functional Enrichment and Conservation Scores of 3-Node Patterns

**Figure 3:**
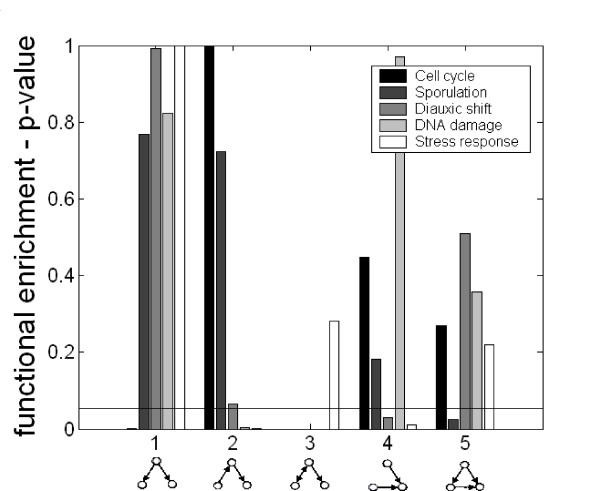
Functional enrichment of all 3-node patterns in each dynamic regulatory network. For each 3-node pattern in each condition we show the p-value obtained in a t-test comparing the *mean functional enrichment score *of the patterns with the corresponding score obtained for the remaining patterns (Methods). Patterns in specific conditions with p-value below 0.05 (marked with a horizontal line) are considered functionally enriched.

We searched the dynamic networks for functionally enriched patterns that may represent design principles underlying specific regulatory mechanisms. For each pattern in each dynamic network we computed the *mean functional enrichment *score that measures the tendency of genes composing an instance of the pattern to have the same GO annotation (Methods, Figure 3). We found that pattern 2 is functionally enriched in DNA damage and stress response conditions while pattern 4 is enriched in diauxic shift and stress response conditions, which are all exogenous conditions. In addition, patterns 1 is enriched in the endogenous cell cycle condition, while pattern 5 in enriched in the endogenous sporulation condition. Examining the functional enrichment of the over-represented network patterns in the various dynamic networks revealed no significant correlation between the two measures. That is, pattern number 5 which is significantly over-represented in 3 dynamic networks is functionally enriched only in a single dynamic network, which is not statistically significant (hyper-geometric p-value = 0.28)

**Figure 4:**
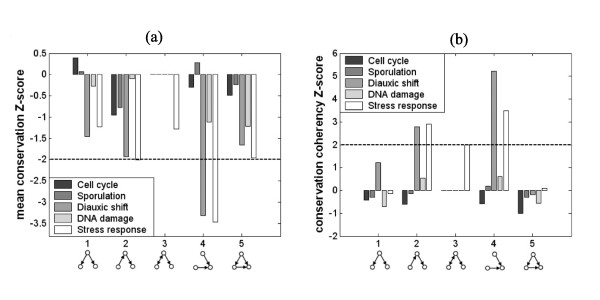
Mean conservation Z-score of all 3-node patterns in each dynamic regulatory networks. Pattern 4 has significantly low *mean conservation score *in diauxic shift and stress response conditions. Pattern 2 has significantly low score in stress response condition. (b) Conservation coherency Z-score of all 3-node patterns in each dynamic regulatory networks. Patterns 2 and 4 have significantly high *conservation coherency score *in diauxic shift and stress response conditions.

We computed the *mean conservation score *and *conservation coherency score *for all patterns in each dynamic network (Methods, Figure 4). Pattern 4 has a significantly low *mean conservation score *in both diauxic shift and stress response dynamic networks, and patterns 2 has a significantly low score in the stress response dynamic network. The significantly low *mean conservation score *for these patterns suggest that the genes composing these patterns are recently evolved. The same patterns, 2 and 4, have significantly high *conservation coherency scores *in the diauxic shift and stress response dynamic networks.

A high *conservation coherency score *indicates that the genes composing the pattern's occurrences tend to have similar conservation score. Specifically, 96% of the occurrences of patterns 2 and 4 in these conditions have a coherent conservation score of zero, representing 3 genes that do not have orthologs in the direct ancestors of *S. cerevisiae*. The fact that no pattern is coherently conserved for longer timescales is surprising considering the fact that 15% of the regulatory genes have conservation scores higher than 1. This may suggest that although some of the regulatory genes of *S. cerevisiae *are highly conserved through evolution there is no evolutionary pressure to maintain their pattern of interactions prior to the direct ancestors of *S. cerevisiae*. This observation is in agreement with previous work by [15] where it was shown that occurrences of motifs in the regulatory networks of *E. coli *and *S. cerevisiae *are not likely to have evolved from the same ancestral pattern by successive duplications. Instead, interaction patterns have converged independently by the interaction of unrelated genes in a process dubbed "convergent evolution".

Examining the conservation of functionally enriched network patterns we find that functionally enriched patterns have significantly low *mean conservation score *and significantly high *conservation coherency score *(Z-score *> *2). These results suggest that specific patterns (such as patterns 2 and 4 in the exogenous diauxic shift and stress response conditions) may represent functional components. However, the structure of such functional components may not be conserved through evolution for long timescales as we do not find highly conserved, functionally enriched patterns. The distinction between regulatory mechanisms operating in endogenous (cell cycle and sporulation) and exogenous (diauxic shift, DNA damage, and stress response) conditions conforms with previous results showing different topological properties in these conditions [11].

## Conclusion

To find whether localized network patterns constitute essential design principles of the underlying network we studied functional enrichment and evolutionary conservation of patterns occurrences using large-scale gene annotation and conservation data. We find some functionally enriched network patterns in different dynamic regulatory networks, supporting the view that network patterns may play a functional role. The same functionally enriched patterns have significantly high *conservation coherency scores *and significantly low *mean conservation scores *in exogenous conditions, representing recently evolved functional components. In a similar analysis of the static regulatory network consisting of all regulatory interactions we found no statistically significant correlation between functionally enriched and recently evolved patterns (data not shown).

Over-representation of network patterns as a method for finding functionally significant network components has attracted significant attention in the system biology community and their examination is of prime interest. Previously, [10] have claimed that over-represented network patterns (motifs) do not play a central regulatory role by examining a small number of specific well-known biological modules. Our results extend their findings showing that over-represented network patterns do not tend to be functionally enriched or evolutionary conserved and thus may not represent significant functional components.

Specifically, we find that the over-represented pattern 5 (feed-forward motif) is not functionally enriched (in all but one dynamic condition) or conserved. However, over-representation of network patterns may become valuable if the correct background model is used to generate random naturally evolved networks. Having found significant network patterns using functional enrichment and conservation criteria, it may be possible to use reverse-engineering techniques to find a correct background model under which over-represented patterns would represent significant modules.

## Methods

### Network data acquisition and processing

We have used the regulatory network of the yeast *S. cerevisiae*, which encompasses all known regulatory interactions constructed from genetic, biochemical and ChIP experiments [11,14]. The static network consists of 3420 nodes representing target genes and 142 nodes representing transcription factors. It contains 7074 directed edges representing regulatory interactions between transcription factors and target genes, or between two transcription factors. All dynamic networks were obtained from [11], which pruned the static regulatory network based on gene expression measurement using the "trace-back" algorithm. The number of genes in the various dynamic networks is significantly lower than that of the static network and is between 286 and 783. Accordingly, the number of regulatory interactions ranges between 481 and 1217.

### Patterns occurrences and over-representation

Patterns occurrences were found using the subgraph isomorphism algorithm of [16]. Over-representation of network patterns is determined by comparing their abundance to the distribution of corresponding values found in an ensemble of randomized networks [3]. The randomized networks preserve each node's incoming and outgoing degree distribution as well as the number of bidirectional edges. For each pattern we computed a Z-score representing the difference between the observed abundance of the pattern in the network and the expected abundance in the randomized networks, divided by the standard deviation of the abundances in the randomized networks.

### Genes conservation scores

Evolutionary retention rates of yeast proteins were previously calculated in [17] by counting for each protein the number of substitutions per amino acid site in orthologous sequences from 21 fully annotated genomes. An alternative approach was used by [18] estimating the propensity of genes to be lost in evolution (PGL) based on a phylogenetic tree consisting of 7 eukaryotes, taking into account the available time estimates for each divergence point. To measure the evolutionary conservation of *S. cerevisiae *genes we defined the evolutionary conservation score based on a phylogenetic tree consisting of 215 species (with no divergence point estimates). Concentrating on *S. cerevisiae *evolution we defined the conservation score as the number of *S. cerevisiae *ancestors that are most likely to contain identifiable orthologs. For that we used the KEGG Sequence Similarity Database (SSDB) [19,20] to list probable orthologs of *S. cerevisiae *genes in all 215 fully sequenced genomes in the database. KEGG's SSDB uses a Smith-Waterman dynamic programming algorithm with a cut-off threshold set to 200. For each gene we assigned binary values indicating the existence of an ortholog at these different species, found at the leaves of the phylogenic tree in KEGG. To predict the presence of orthologs in *S. cerevisiae *ancestors we applied the maximum parsimony algorithm PARS from PHYLIP package [21]. We then used the number of ancestors that contain orthologs on the path from *S. cerevisiae *to the root (scoring 0–4) as the evolutionary conservation score. A related method to determine evolutionary conservation pressure was employed in [13], which measured the presence of orthologs in a set of five higher eukaryotes (not considering their phylogeny). We note that there is a statistically significant correlation between our conservation scores and that of [18].

### Network pattern scores

To formulate patterns' scores, we first define *C *: *N *→ {0...4} as the conservation score of single genes, where *N *= {1...*n*} denotes the network's nodes. The *j*th occurrence of pattern *i *is a node triplet denoted by Oji
 MathType@MTEF@5@5@+=feaafiart1ev1aaatCvAUfKttLearuWrP9MDH5MBPbIqV92AaeXatLxBI9gBaebbnrfifHhDYfgasaacH8akY=wiFfYdH8Gipec8Eeeu0xXdbba9frFj0=OqFfea0dXdd9vqai=hGuQ8kuc9pgc9s8qqaq=dirpe0xb9q8qiLsFr0=vr0=vr0dc8meaabaqaciaacaGaaeqabaqabeGadaaakeaacqWGpbWtdaqhaaWcbaGaemOAaOgabaGaemyAaKgaaaaa@30B8@ ∈ *N*^3^, where 1 ≤ *i *≤ 13, 1 ≤ *j *≤ *m*_*i*_, and *m*_*i *_is the total number of occurrences of pattern *i*. Oji
 MathType@MTEF@5@5@+=feaafiart1ev1aaatCvAUfKttLearuWrP9MDH5MBPbIqV92AaeXatLxBI9gBaebbnrfifHhDYfgasaacH8akY=wiFfYdH8Gipec8Eeeu0xXdbba9frFj0=OqFfea0dXdd9vqai=hGuQ8kuc9pgc9s8qqaq=dirpe0xb9q8qiLsFr0=vr0=vr0dc8meaabaqaciaacaGaaeqabaqabeGadaaakeaacqWGpbWtdaqhaaWcbaGaemOAaOgabaGaemyAaKgaaaaa@30B8@(*l*),1 ≤ *l *≤ 3, represents the occurrence's *l*th node.

#### Mean functional enrichment score

Functional enrichment of network patterns is computed based on the enrichment of GO Process annotations in genes composing the different pattern occurrences. Specifically, let *E*(Oji
 MathType@MTEF@5@5@+=feaafiart1ev1aaatCvAUfKttLearuWrP9MDH5MBPbIqV92AaeXatLxBI9gBaebbnrfifHhDYfgasaacH8akY=wiFfYdH8Gipec8Eeeu0xXdbba9frFj0=OqFfea0dXdd9vqai=hGuQ8kuc9pgc9s8qqaq=dirpe0xb9q8qiLsFr0=vr0=vr0dc8meaabaqaciaacaGaaeqabaqabeGadaaakeaacqWGpbWtdaqhaaWcbaGaemOAaOgabaGaemyAaKgaaaaa@30B8@, *t*) denote the probability that a random choice of 3 genes from the network has a higher number of genes annotated with term *t *than the *j*th occurrence of pattern *i*, assuming a hyper-geometric distribution. The *mean functional enrichment score *of pattern *i *is the average *E*(Oji
 MathType@MTEF@5@5@+=feaafiart1ev1aaatCvAUfKttLearuWrP9MDH5MBPbIqV92AaeXatLxBI9gBaebbnrfifHhDYfgasaacH8akY=wiFfYdH8Gipec8Eeeu0xXdbba9frFj0=OqFfea0dXdd9vqai=hGuQ8kuc9pgc9s8qqaq=dirpe0xb9q8qiLsFr0=vr0=vr0dc8meaabaqaciaacaGaaeqabaqabeGadaaakeaacqWGpbWtdaqhaaWcbaGaemOAaOgabaGaemyAaKgaaaaa@30B8@, *t*), where for each occurrence *j *we take the term *t *with the lowest probability.

MESi=1mi∑j=1mimin⁡t E(Oji,t)
 MathType@MTEF@5@5@+=feaafiart1ev1aaatCvAUfKttLearuWrP9MDH5MBPbIqV92AaeXatLxBI9gBaebbnrfifHhDYfgasaacH8akY=wiFfYdH8Gipec8Eeeu0xXdbba9frFj0=OqFfea0dXdd9vqai=hGuQ8kuc9pgc9s8qqaq=dirpe0xb9q8qiLsFr0=vr0=vr0dc8meaabaqaciaacaGaaeqabaqabeGadaaakeaacqWGnbqtcqWGfbqrcqWGtbWudaahaaWcbeqaaiabdMgaPbaakiabg2da9maalaaabaGaeGymaedabaGaemyBa02aaSbaaSqaaiabdMgaPbqabaaaaOWaaabCaeaadaWfqaqaaiGbc2gaTjabcMgaPjabc6gaUbWcbaGaemiDaqhabeaaaeaacqWGQbGAcqGH9aqpcqaIXaqmaeaacqWGTbqBdaWgaaadbaGaemyAaKgabeaaa0GaeyyeIuoakiabbccaGiabdweafjabcIcaOiabd+eapnaaDaaaleaacqWGQbGAaeaacqWGPbqAaaGccqGGSaalcqWG0baDcqGGPaqkaaa@4EDB@

To assess the statistical significance of a pattern's *mean functional enrichment score *we compare it to a *mean functional enrichment score *obtained for the occurrences of the remaining 3-node patterns using a t-test.

#### Mean conservation score

The *mean conservation score *of pattern *i *is the average conservation score of all nodes in the pattern's occurrences,

MCSi=13mi∑j=1mi∑l=13C(Oji(l))
 MathType@MTEF@5@5@+=feaafiart1ev1aaatCvAUfKttLearuWrP9MDH5MBPbIqV92AaeXatLxBI9gBaebbnrfifHhDYfgasaacH8akY=wiFfYdH8Gipec8Eeeu0xXdbba9frFj0=OqFfea0dXdd9vqai=hGuQ8kuc9pgc9s8qqaq=dirpe0xb9q8qiLsFr0=vr0=vr0dc8meaabaqaciaacaGaaeqabaqabeGadaaakeaacqWGnbqtcqWGdbWqcqWGtbWudaahaaWcbeqaaiabdMgaPbaakiabg2da9maalaaabaGaeGymaedabaGaeG4mamJaemyBa02aaSbaaSqaaiabdMgaPbqabaaaaOWaaabCaeaadaaeWbqaaiabdoeadjabcIcaOiabd+eapnaaDaaaleaacqWGQbGAaeaacqWGPbqAaaGccqGGOaakcqWGSbaBcqGGPaqkcqGGPaqkaSqaaiabdYgaSjabg2da9iabigdaXaqaaiabiodaZaqdcqGHris5aaWcbaGaemOAaOMaeyypa0JaeGymaedabaGaemyBa02aaSbaaWqaaiabdMgaPbqabaaaniabggHiLdaaaa@5084@

To assess the statistical significance of a *mean conservation score *we computed a Z-score comparing it to the expected *mean conservation score *under a random permutation of conservation scores of genes in the network. Specifically, for a given permutation *σ *of the network's nodes, we computed the MCS using C¯
 MathType@MTEF@5@5@+=feaafiart1ev1aaatCvAUfKttLearuWrP9MDH5MBPbIqV92AaeXatLxBI9gBaebbnrfifHhDYfgasaacH8akY=wiFfYdH8Gipec8Eeeu0xXdbba9frFj0=OqFfea0dXdd9vqai=hGuQ8kuc9pgc9s8qqaq=dirpe0xb9q8qiLsFr0=vr0=vr0dc8meaabaqaciaacaGaaeqabaqabeGadaaakeaacuWGdbWqgaqeaaaa@2DD3@(*i*) = *C*(*σ*(*i*)), instead of *C*(*i*). This background model, preserving the topology of the original network, was also used by [13].

#### Conservation coherency score

The *conservation coherency score *for pattern *i *is defined as the percentage of the pattern's occurrences with all nodes having the same conservation score:

CCSi=1mi|{j|1≤j≤mi,∀1≤x,y≤3C(Oji(x))=C(Oji(y))}|
 MathType@MTEF@5@5@+=feaafiart1ev1aaatCvAUfKttLearuWrP9MDH5MBPbIqV92AaeXatLxBI9gBaebbnrfifHhDYfgasaacH8akY=wiFfYdH8Gipec8Eeeu0xXdbba9frFj0=OqFfea0dXdd9vqai=hGuQ8kuc9pgc9s8qqaq=dirpe0xb9q8qiLsFr0=vr0=vr0dc8meaabaqaciaacaGaaeqabaqabeGadaaakeaacqWGdbWqcqWGdbWqcqWGtbWudaahaaWcbeqaaiabdMgaPbaakiabg2da9maalaaabaGaeGymaedabaGaemyBa02aaSbaaSqaaiabdMgaPbqabaaaaOGaeiiFaWNaei4EaSNaemOAaOMaeiiFaWNaeGymaeJaeyizImQaemOAaOMaeyizImQaemyBa02aaSbaaSqaaiabdMgaPbqabaGccqGGSaalcqGHaiIidaWgaaWcbaGaeGymaeJaeyizImQaemiEaGNaeiilaWIaemyEaKNaeyizImQaeG4mamdabeaakiabdoeadjabcIcaOiabd+eapnaaDaaaleaacqWGQbGAaeaacqWGPbqAaaGccqGGOaakcqWG4baEcqGGPaqkcqGGPaqkcqGH9aqpcqWGdbWqcqGGOaakcqWGpbWtdaqhaaWcbaGaemOAaOgabaGaemyAaKgaaOGaeiikaGIaemyEaKNaeiykaKIaeiykaKIaeiyFa0NaeiiFaWhaaa@6821@

Specifically, for each 3-node pattern occurrence, we test the conservation coherency, i.e., whether all 3 nodes have the same conservation score. To assess the statistical significance of this score, we used a similar random permutation background model as for the *mean conservation score*.

## Authors' contributions

OM and TS analyzed the data. OM, TS and ER wrote the paper.
